# Fracture nonunion and delayed union

**DOI:** 10.1016/j.jposna.2024.100058

**Published:** 2024-04-09

**Authors:** David S. Liu, Brian D. Snyder, Susan T. Mahan

**Affiliations:** 1 Harvard Combined Orthopaedic Residency Program, Boston, MA, USA; 2 Department of Orthopaedics, Boston Children’s Hospital, Boston, MA, USA; 3 Department of Orthopaedic Surgery, Boston Children’s Hospital, Boston, MA, USA

**Keywords:** Bone health, Vitamin D, Fracture nonunion, Pediatric fracture

## Abstract

Delayed union and nonunion of fractures exist in the pediatric population. Fracture healing requires the synergistic collaboration of mechanical support and robust biological processes to allow endochondral ossification, reestablishment of bone continuity, and subsequent remodeling to strong lamellar bone. Failure of either mechanical stability or biology may manifest as delayed fracture healing. While early recognition of potential metabolic and pharmacologic risk factors may be addressed by pre-emptive treatment using nutritional and vitamin D supplements, definitive treatment of established nonunion requires a comprehensive approach.

**Key Concepts:**

(1) Delayed unions and nonunions are more common in adult bone but can also occur in pediatric bone.
(2) Fracture healing requires synergistic collaboration of mechanical support and robust biological processes; treatment of nonunions should address both the biological and mechanical factors.
(3) Vitamin D is an integral component of calcium absorption and bone health.

## Introduction

In pediatric fracture care, the common belief is that fractures always heal and that patients return to preinjury activity without functional limitations. While it is true that the majority of fractures sustained by healthy children and young adults can often be successfully treated with closed reduction and cast immobilization, there are some that need surgical intervention. A small but significant percentage of fractures can result in delayed union (4-6 months) or even nonunion (9-12 months). Surgical treatment of pediatric fractures is often performed for fracture patterns and circumstances thought to increase the risk for delayed or nonunion as a consequence of intrinsic structural instability (large displacement, multiple fragments) and/or factors that affect the biology of fracture healing (open, contaminated wound or infection, extensive violation soft tissue envelope, medical comorbidities, habits or medications). To estimate the risk factors for delayed fracture healing in children and young adults, providers must understand the integral dependence of fracture healing on bone structure and health, which also informs treatment strategies.

## Bone structure and health

Bone is a living structure comprised of an organic matrix (osteoid), calcium hydroxyapatite crystals, and noncollagenous proteins. The osteoid becomes mineralized with deposits of calcium and phosphate, conferring compressive stiffness, while the collagen I fibril structural matrix provides tensile strength to the bone tissue scaffold. The stiffness and strength of bone depend on the mineral density of the bone tissue and the geometry (shape) of the bone, represented by the cross-sectional area and moment of inertia [1]. The moment of inertia quantifies how the bone tissue is distributed in space; it varies as the fourth power of the distance of the bone tissue relative to a specific bending axis. The resistance of the bone to bending and torsion dramatically increases as bone tissue is distributed away from that bending axis (eg, periosteal expansion of fracture callus augments fracture stability by using geometry to compensate for weak woven bone), and conversely is severely diminished for narrow, gracile bones (eg, nonambulatory patients). Mineralized bone tissue is stiffest and strongest in compression, weaker in tension and weakest in shear. Torsional moments that induce tensile and shear stresses within the bone tissue will cause the bone to fracture before axially applied compressive loads. It is the *least rigid* segment through the bone that dictates the load capacity of the entire bone; that is, fracture occurs at the segment through the bone with the lowest combined bone mineral tissue density and cross-sectional geometry.

An active organ, the skeleton is constantly remodeling even after the completion of statural growth. Fracture repair via interfragmentary endochondral ossification recapitulates the mechanisms of normal bone remodeling where bone formation is mediated by osteoblasts, bone resorption is mediated by osteoclasts, the temporal and spatial sequence of initial bone resorption during the inflammatory (mesenchymal) phase of fracture repair tightly coordinated with the subsequent bone formation during the repair (bridging chondroid/soft callus followed by chondroid-osteoid/hard callus) and remodeling (osteogenic) phases, regulated by local cytokines and circulating hormones, including vitamin D, parathyroid hormone (PTH), insulin-like growth factor, and calcitonin. As such, osseous blood flow is the major determinant of fracture healing, requisite to deliver nutrients, growth factors, hormones, inflammatory (type I and II macrophage), mesenchymal and osteoprogenitor cells to the site of bone injury.

Bone health and the antecedents of osteopenia are established in childhood and adolescence. When bone resorption exceeds bone formation, there is a net decrease in mineralized bone tissue volume resulting in osteopenia. Increased bone fragility is associated with osteopenia (decreased bone mineral density), the manifestation of either osteoporosis (normal mineralization of osteoid, but decreased mineralized tissue volume) or osteomalacia (hypo-mineralized osteoid). Osteoporosis is a major cause of morbidity and excessive medical resource utilization worldwide, especially in postmenopausal women. Thought only to be part of aging, osteoporosis is now considered to have its roots in childhood; the bone mass attained in childhood is an important modifiable determinant of lifelong skeletal health, with peak bone mass achieved before age 30, declining thereafter [2]. Proper diagnostic studies to determine the cause of osteopenia are required because osteoporosis is treated differently than osteomalacia (the consequence of vitamin D deficiency, poor nutrition, malabsorption, anticonvulsant drug induction of p450 micro-enzymes, renal or hepatic failure, steroid administration or endocrinopathy). Patients at risk for osteomalacia may benefit from dietary modifications to increase calcium and vitamin D intake, whereas patients with osteoporosis may benefit from the administration of bisphosphonates to improve bone volume by decreasing osteoclastic bone resorption.

### Calcium

Approximately 99% of the total body calcium resides in bone. Besides being a ubiquitous cation essential for intracellular cell signaling, calcium is a requisite inorganic component of bone tissue mineralization. Dietary consumption of calcium is vital to maintaining homeostasis during growth, fracture healing, and bone remodeling. The Institute of Medicine has recommended the dietary intake of calcium and vitamins that meet the requirements of 97.5% of the population (Table 1). For Calcium, 700 mg is recommended for ages 1 to 3, 1,000 mg for ages 3 to 9, 1,300 mg for ages 9 to 18, and 1,000 mg for age >18. In the United States, less than 15% of people meet these recommended daily allowances (RDA). [3].

**Table 1:** Institute of Medicine recommended dietary intake for calcium.

Age	Calcium	
	RDA (mg/d)	UL (mg/d)*
Infants		
0-6 mo	200†	1,000
6-12 mo	260†	1,500
1-3 y	700	2,500
4-8 y	1,000	2,500
9-13 y	1,300	3,000
14-18 y	1,300	3,000

*RDA, recommended daily allowances*.

* Upper limit (UL) indicates the level above which there is a risk for adverse events.

† As RDAs have not been established for infants, these values reflect adequate intake reference.

In children, the routine supplementation of dietary calcium has not been shown to significantly increase bone mineral density and therefore is not generally encouraged, instead, emphasizing a well-balanced diet with calcium intake at or near the RDA [4]. However, for children and adolescents who are unable to consume enough calcium from dietary sources, supplementation may be prescribed. Two common forms of supplemental calcium are calcium carbonate (40% elemental calcium) and calcium citrate (21% elemental calcium). Calcium carbonate must be taken with meals to promote absorption, whereas calcium citrate does not require gastric acid for absorption.

### Vitamin D_3_

Vitamin D_3_ is a fat-soluble hormone necessary for the absorption and utilization of calcium. Its synthesis is a complex process involving multiple organ systems. 7-Dehydrocholesterol (provitamin D), present in the dermal layer of the skin, is converted to cholecalciferol with exposure to UV-B. This compound is modified by hydroxylation in the liver to create calcidiol. Stimulated by the increase of PTH and/or the decrease of calcium and phosphate, calcidiol is further hydroxylated to its active form, calcitriol, in the proximal tubule of the kidney. Calcitriol regulates calcium and phosphate metabolism by: (1) stimulating their release from bone by osteoclastic resorption, (2) by increasing their intestinal absorption, and (3) by increasing their renal reabsorption. In vitamin D deficiency (nutritional rickets) only 10% to 15% of dietary calcium and phosphate are absorbed from the intestine, the resulting hypocalcemia and hypophosphatemia provoke ↑PTH, which activates osteoclasts and promotes the kidney to decrease renal wasting of calcium and increase excretion of phosphate. Overall these changes induce hypo-mineralization of osteoid, (osteomalacia) [5]. While the effect of vitamin D_3_ promoting anabolic bone metabolism is unquestioned in pediatric studies, the predictive value of serum 25(OH)-vitamin D levels (categorized according to thresholds: deficient <20 ng/mL, insufficient 20 to 30 ng/mL, low normal 30 to 40 ng/mL, normal >40 ng/mL) has received much less attention [6], [7], [8], [9]. Cross-sectional investigations of vitamin D status in adolescents revealed deficiency in 17% to 47% of adolescents [10], linked to race, (black and Hispanic), obesity, and the winter season. Based on a retrospective analysis of a large database, Zura et al. observed a pediatric fracture nonunion rate of 0.85%, associated with vitamin D deficiency [11]. The recommended RDA for vitamin D intake is not uniform and varies by organization (Table 2) [12].

**Table 2:** Various RDA recommendations for Vitamin D.

Institute of medicine	
Age (years)	Vitamin D recommendation (IU/Day)
<1	400
1-80	600
≥80	800

Endocrine society	
Age (years)	Vitamin D recommendation (IU/Day)
<1	400-1,000
≥1	800-4,000

Pediatrics to geriatrics in Orthopaedics instructional course lecture	
Age (years)	Vitamin D recommendation (IU/Day)
0-1	400
1-8	600-1,000
8-13	1,000-1,500
13-18	1,500-2,000
Adults	2,000

Weight (lbs)	Vitamin D recommendation (IU/Day)
20-50	500-1,000
50-90	1,500
90	2,000

*RDA, recommended daily allowances*.

Although vitamin D toxicity is rare, the health effects can be serious. There were 11,718 cases of excess vitamin D exposure recorded in the National Poison Data System caused by inadvertent or intentional excess intake of vitamin D, with more than half of the cases in children <5 years [13]. Endogenous causes also exist, related to extra production of active vitamin D metabolites (ie, Williams-Beuren syndrome, chronic granulomatous disorders, or some lymphomas) or reduced degradation of metabolites (ie, idiopathic infantile hypercalcemia). Clinical presentations are manifestations of the resultant hypercalcemia: symptoms of confusion, apathy, recurrent vomiting, abdominal pain, polyuria, polydipsia, and dehydration. Administering extremely large doses of cholecalciferol, 240,000 to 4500,000 IU, resulted in serum 25-hydroxyvitamin D levels of 250 to 670 ng/mL leading to severe hypercalcemia [14].

## Delayed union and nonunion

### Risk factors

**Figure 1:**
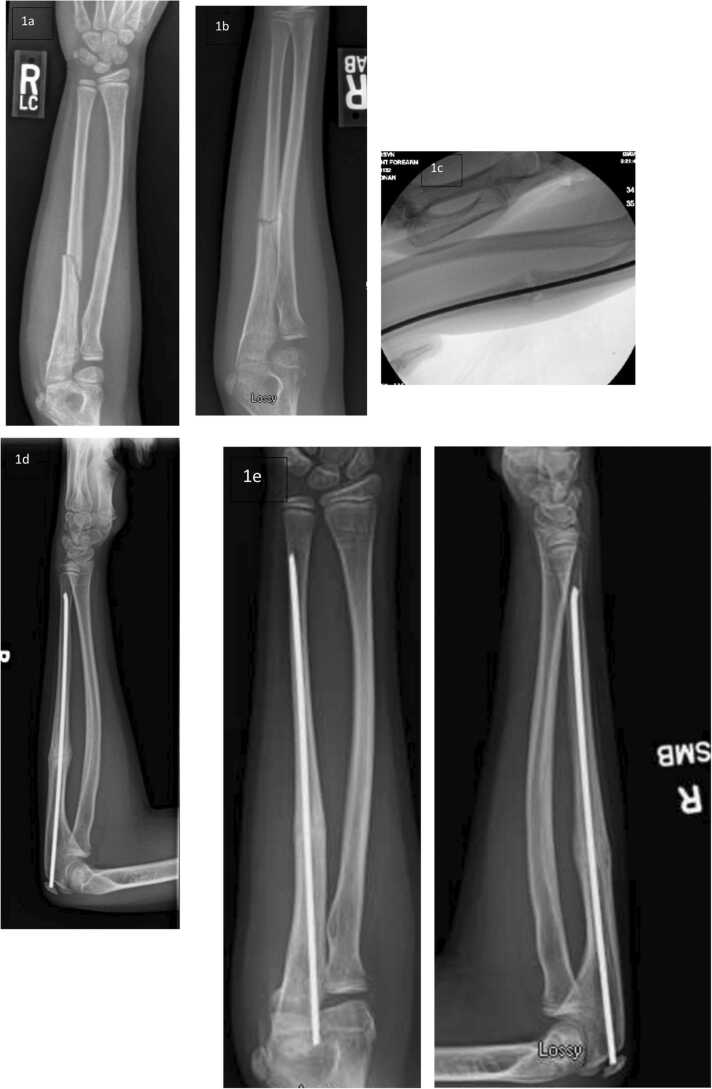
KM is an 8.5-year-old gymnast who fell of the uneven bars and broke her R ulna (Fig. 1a). She suffered a refracture 4 months later and was treated with a series of casts and splints at 7 months she had persistent lucency and pain (Fig. 1b). Metabolic work revealed slightly low vitamin D level with hypercalciuria of unknown etiology. She was treated vitamin D, Calcium and underwent IM fixation of her ulna (Fig. 1c). She was subsequently seen in genetics 1 year after injury and was diagnosed with a defect in Type 2 collagen formation. Her ulna was healing at 21 months (Fig. 1d) and by 26 months was finally healed (Fig. 1e). The family and treating surgeons elected to leave the implant in. Case example courtesy of Kenneth Noonan MD MHCDS. Fig. 1a. Isolated ulna fracture in 8-year healthy gymnast. Fig. 1b. 6 months after the injury she had persistent fracture lucency after suffering a refracture Fig. 1c. IM fixation was performed at 7 months from original injury. Fig. 1d. 21 months after the fracture her ulna has periosteal bridging and a slight lucency at fracture site. Fig. 1e. 26 months after her original fracture she is fully healed.

Fractures are common within the pediatric population, accounting for 10% to 25% of all pediatric injuries [15]. See Fig. 1. The majority of fractures sustained by healthy children and young adults heal rapidly with the uncomplicated restoration of skeletal anatomy; however, when fracture healing mechanisms fail, delayed union is the consequential outcome [16]. In the adult population, 5% to 10% for long bone fractures go on to nonunion, while in the pediatric population, nonunion is rare, reported to be 0.85% in a study of >200,000 pediatric fractures [17]. The definition for nonunion with regard to timing is challenging to define. The definition of nonunion put forth by the FDA is a fracture that persists for a minimum of 9 months without signs of healing for 3 months [18]. However, the definition of nonunion does vary based on fracture location and age; for example, a pediatric lateral condyle fracture is considered nonunited as early as 8 weeks [19]. Risk factors for nonunion are related to the mechanical and biological factors that influence fracture healing: fracture severity (pattern ± bone loss), anatomic location and type of bone (cortical vs cancellous), mechanism of injury/energy imparted, viability soft tissue envelope (the integrity of osseous blood supply), wound contamination (open fractures), functional status (sarcopenia, disuse osteopenia), medical comorbidities (diabetes, endocrinopathy, nephropathy, hepatopathy, vasculopathy, neuropathy), nutritional status (nitrogen balance, vitamin D deficiency), habits (smoking/nicotine, alcohol, recreation drugs) and medications (steroids, diuretics, antiepileptics, opioids, anticoagulants) [17], [20]. The metaphysis comprised of low density, porous trabecular bone is intrinsically weaker but metabolically more active than the dense cortical bone comprising the diaphysis; owing to a less robust intraosseous blood supply, the diaphysis is frequently the site of fracture nonunions. Fractures encompassing the tibial diaphysis, femoral neck, and scaphoid where the vascular supply is tenuous have the highest risk of nonunion. As a consequence of disruption of the radial artery branch dorsal ridge blood supply, scaphoid fractures involving the proximal third demonstrate the same nonunion rates in adolescents as in adults. Nonunion after lateral humeral condyle fractures is another well-known complication of intra-articular synovial joint fractures, resulting from inhibition of hematoma/fibrin clot formation during the inflammatory phase of fracture healing due to the constant bathing of the fracture site by synovial fluid.

Successful fracture healing requires the synergistic collaboration of mechanical support to restore and maintain skeletal anatomy and to provide sufficient stability across the fracture site to facilitate a cascade of biological processes that initiate and form fracture callus, instigate progressive endochondral/intramembranous ossification of the fibrocartilaginous callus that re-establishes bone continuity and subsequently activates Haversian remodeling to rebuild strong lamellar bone. Delayed fracture healing represents either a failure of mechanics to sufficiently stabilize and reconstitute the disrupted bone anatomy or a failure of biology to propagate the fracture healing cascade, or a combination of both. Hypertrophic nonunions (abundant callus formation with unmineralized fibrocartilage bridging the fracture site) are a manifestation of inadequate fracture reduction and/or mechanical immobilization of the fracture fragments, with preservation of an adequate blood supply. In this case, endonchondral ossification failed to replace the bridging soft callus by woven bone (predominance type II rather than type I collagen). Atrophic or oligotrophic nonunions (little or no callus formation) are the result of inadequate fracture reduction/mechanical immobilization of the fracture fragments *and* insufficient restoration of the osseous blood supply.

Failures of biology may be inherent to the underlying pathophysiology contributing to the fracture occurrence (eg, tibial pseudoarthrosis in children with neurofibromatosis type 1 [21], which manifests abnormal vascularization of skeletal tissues, reduced proliferation, and differentiation of chondrocytes that culminate in poor quality bone or osteogenesis imperfecta, where abnormal COL 1 synthesis creates intrinsically abnormal mineralized bone tissue) [22]. Rheumatologic diseases are associated with osteoporosis, increased fracture risk, and delayed fracture healing secondary to chronic glucocorticoid exposure [23]. Hypothyroidism and growth hormone deficiencies affect skeletal growth at the physis via endochondral ossification that can also influence fracture repair [24]. However, most nonunions are related to modifiable etiologies as a consequence of dietary and/or environmental factors such as poor diet/nutrition with low calcium and vitamin D intake, childhood obesity, environmental exposures such as lead, heavy metals, chemical toxins as well as teenage smoking/vaping (nicotine and carbon monoxide) contributing to poor bone health in children [25], [26]. For pediatric fractures, vitamin D deficiency has been linked to increased fracture risk [27], need for surgical fixation [28] and the threat of delayed union [17]. Furthermore, vitamin D deficiency may amplify both the craving for and the effects of opioid medications, thereby exacerbating addictive behavior [29]. Studies have suggested a relationship between opioids and nonunion, though more research is needed [30]. Based on experimental and clinical evidence in vitamin D deficient and insufficient children, vitamin D_3_ supplementation promotes fracture healing [31], [32], [33].

Medication use has been associated with an increased risk of nonunion in children and adolescents. Chronic exposure to supraphysiologic glucocorticoids has been linked to reduced bone mineral density, increased fracture rates, and compromised statural growth [34]. Glucocorticoids have been implicated in delayed union and nonunion of pediatric fractures by decreasing callus proliferation. Contraceptive and reproductive steroid use has been connected to increased nonunion rates in adolescents [17]. Nonsteroidal anti-inflammatory drugs (NSAIDs) have been [35], [36] incriminated to increase fracture nonunion rates in adults, predicated on the effects of NSAIDs on prostaglandins that mediate osteoclastic bone resorption and osteoblastic bone formation. During the inflammatory phase of fracture repair, prostaglandins are synthesized from arachidonic acid, a reaction catalyzed by cyclooxygenase (COX) enzyme. Traditional NSAIDs and selective COX-2 inhibitors interfere with upregulation of COX-2, and prostaglandin synthesis. However, multicenter retrospective studies and meta-analyses have substantiated that NSAID exposure in children does not increase the risk for nonunion [37], [38], [39]. Ibuprofen has been shown to be an effective analgesic that does not impair fracture healing in the skeletally immature patient [40], [41], [42]. Bisphosphonates are commonly used for patients with genetic (eg, osteogenesis imperfecta), or acquired (eg, metastatic cancer) metabolic bone diseases that produce generalized or localized deficits in bone mineralization, heterotopic calcification, and/or hypercalcemia. Bisphosphonates bind via zinc ligands to hydroxyapatite and block bone resorption by inhibiting farnesyl pyrophosphate synthase, important for promoting the attachment of osteoclasts to bone, thereby preventing diminution of bone mineral density and mitigating fragility fractures [43]. Studies in postmenopausal females on long-term bisphosphonate therapy revealed an increased incidence of atypical femoral neck (fatigue) fractures [44], [45], [46], [47]. In adults, bisphosphonates have been implicated with delayed fracture healing [48] and prolonged healing times [49] by hindering the remodeling of hard callus/woven bone to lamellar bone (reduced biomarkers of resorption and synthesis) [50]. However, data from level 1 clinical trials indicate that initiating bisphosphonates as early as 2 weeks postfracture does not increase rates of nonunion or malunion [51].

In the setting of infection, the ability for bone healing is a complex balance between infectious load, specific organisms, treatment types, infection location, and fracture characteristics and biomechanics [52]. The majority of fracture-related infections are bacterial in origin, mostly *Staphylococcus aureus* or polymicrobial infections [53]. At a molecular level, *S aureus* uses surface proteins called microbial surface components recognizing adhesive matrix molecules to adhere to collagen and fibronectin on bone, allowing for biofilm formation, a protective layer of polysaccharides with bacteria that grow on bone and foreign bodies (such as implants). When infection is present at the ends of fracture fragments, bone healing is compromised, which results in infected fracture nonunions. Failure to clear infection causes continued bone and tissue destruction, which may lead to draining sinus tracts, bone abscesses, and failed fracture fixation [54], [55], [56], [57], [58], [59], [60]. In cases of suspected delayed fracture union or nonunion, a comprehensive workup should be initiated. The modified Radiographic Union Score for Tibial Fractures allows for the characterization of bridging callus and demonstrates a high degree of intraobserver and interobserver reliability in the evaluation of fracture healing. Although it was initially developed for the assessment of tibia fractures, modified Radiographic Union Score for Tibial has been used to evaluate healing in a wide variety of fractures [54], [55], [56], [57], [58], [61]. While common inflammatory markers including white blood count, erythrocyte sedimentation rate, and C-reactive protein may be elevated in acute infection, these markers have not been shown to be predictive of infectious nonunion [62].

### Treatment

Noninvasive adjunctive therapies have been demonstrated to be effective for treating delayed unions. Low-intensity pulsed ultrasound accelerates fracture healing and increases the strength of the evolving fracture callus via integrin mechano-receptors involved in cellular signaling and osteogenic differentiation [63]. Mesenchymal cells translate the micro-mechanical signal to a biochemical response, increasing expression of early osteogenic genes (osteonectin, osteopontin, IGF-1) and induce intracellular signaling in osteoblasts to activate COX-2, which in turn stimulates production of prostaglandin E2, critical to endochondral ossification of the soft callus [64]. Electrical stimulation to facilitate fracture healing has been used for >4 decades [65]. The applied electrical field induces stress-generated potentials by piezoelectric effects (applied voltage causes mechanical deformation in the tissue) and streaming potentials (electrically charged anions and cations flowing through a channel/pore creates a current or charge separation that produces transmembrane potentials). Direct current electrical stimulation requires percutaneous insertion of a cathode into the fracture site and an anode in the adjacent soft tissue to create a unidirectional current that produces an alkaline, hypoxic micro-environment that stimulates osteoclasts to produce VEGF, which promotes angiogenesis. Capacitive coupling alternating current generators create an electrical field via surface electrodes placed across the fracture site that affect voltage-gated calcium channel signaling in osteoblasts related to upregulation of BMP2 and 4 and TGF-β1, which promote collagen synthesis and calcification of fracture callus during enchondral ossification. Similarly, pulsed electromagnetic fields applied across the fracture site are postulated to increase cytoplasmic calcium by activating intracellular voltage-gated calcium channels that are associated with the upregulation of key growth factors (IGF-2, BMP 2 and 4, TGF-β1) that induce osteoblast differentiation, proliferation, and extracellular matrix deposition.

Management of mechanical instability requires fracture reduction to restore skeletal anatomy, stabilized by interfragmentary compression produced by either external fixators (eg, Illizarov ring fixators) or rigid internal fixation using compression plates or intramedullary nails while preserving a viable intraosseous blood supply. Excision of sclerotic margins along the nonunion interface restores communication with the intramedullary canal bone marrow to promote the migration of mesenchymal and osteoprogenitor cells.

Failed bone biology can be multi-factorial, requiring a comprehensive approach. In dysvascular bone, the consequence of a devitalized soft tissue envelope, vascularized muscle flaps or vascularized bone grafts may be required to reconstitute a functional blood supply to promote angiogenesis and restore blood flow to the fracture site. When there is infection (osteomyelitis), a 2 stage approach is recommended: stage 1—remove infected implants, aggressively debride infected/avascular tissues, administer local (antibiotic impregnated calcium sulfate resorbable beads) and systemic antibiotics based on tissue-based culture and sensitivities; stage 2 (after definitive eradication of infection)—attain rigid interfragmentary mechanical stabilization ± bone grafting (autogenous cancellous bone) ± fracture repair enhancers (electrical stimulation, low-intensity pulsed ultrasound, demineralized bone matrix, biologics-BMP-2). For large segmental defects Masquelet technique or bone transport using distraction osteogenesis may be necessary. Autogenous cancellous bone graft, typically harvested from the iliac crest remains the gold standard for treating nonunions in that it is osteoconductive, providing a structural scaffold to support osteogenesis; it is osteoinductive, providing growth factors such as bone morphogenetic protein (BMP) (promotes osteogenic differentiation of mesenchymal stem cells, upregulates osteogenic lineage genes osterix and osteocalcin as well as stimulates angiogenesis), TGF-β (regulates cartilage and bone formation of fracture callus by inducing mesenchymal cells to produce COL2 and proteoglycans as well as osteoblasts to synthesize COL1), IGF-II (stimulates cellular proliferation, cartilage matrix and COL1 synthesis), and PDGF (chemotaxis-attracts inflammatory cells to the fracture site); it directly supplies osteogenic cells (mesenchymal stem cells, osteoblasts and osteocytes) requisite to initiate and propagate fracture repair [66]. However, because of the associated donor site morbidity incurred when harvesting the autograft, alternative strategies have been employed. Structural allograft and synthetic bone graft material are only osteoconductive but may be augmented with bone marrow aspirate to provide osteogenic cells and growth factors. Demineralized bone matrix, comprised of a composite of collagens (mostly type I, with some type IV and X), a variable amount of calcium phosphate, noncollagenous proteins, and growth factors (including several BMPs) is osteoinductive, but has poor structural integrity for osteoconduction. Recombinant BMP-2 administered to fracture nonunions during revision surgery has been demonstrated to enhance healing. However, providers should be aware that the use of BMP-2 has been approved by the FDA only for tibial nonunions and anterior lumbar interbody spinal fusion, but surgeon-directed use in pediatric nonunions as a consequence of an underlying bone pathology has been associated with a low-risk profile [67].

## Conclusion

Delayed and nonunion of fractures do exist in pediatric fractures, though at a significantly lower rate than the adult population. Successful fracture healing requires the synergistic collaboration of mechanical support to restore skeletal anatomy and to provide sufficient stability across the fracture site to facilitate a cascade of biological processes that initiate with angiogenesis and progress to endochondral ossification to re-establish bone continuity and subsequent remodeling to strong lamellar bone. Delayed fracture healing represents either a failure of mechanics to sufficiently stabilize and reconstitute the disrupted bone anatomy or a failure of biology to promulgate the temporal and spatial sequence of bone repair regulated by local cytokines and circulating hormones, including vitamin D, PTH, IGF, BMP, TGF-β and calcitonin. Recognition of potential metabolic and pharmacologic risk factors that prevent effective fracture repair may be addressed by pre-emptive treatment using nutritional and vitamin D supplements (Table 3). Definitive treatment of established nonunion requires a comprehensive approach.

**Table 3:** Treatment goals and options for delayed union and nonunion.

Increase stability
• Surgical stabilization (external or internal fixation)
• Prolonged cast immobilization
Promote biology
• Nutritional supplementation (Vitamin D, calcium)
• Low intensity pulsed ultrasound
• Direct current electrical stimulation
• Osteoconductive agents (autogenous bone graft, allogenic bone graft, or synthetic bone graft)
• Osteoinductive agents (autogenous bone graft, bone growth factors (BMP, TGF-β, IGF-II, PDGF)
• Eradicate infection in setting of infectious nonunion.

BMP, bone morphogenetic protein.

## Author contributions

**Brian D. Snyder:** Writing – review & editing. **David S. Liu:** Validation, Writing – original draft. **Susan T. Mahan:** Writing – review & editing.

## Declarations of competing interests

The authors declare the following financial interests/personal relationships which may be considered as potential competing interests: David Liu, MD, reports financial support was provided by Orthopaedic Research and Education Foundation. If there are other authors, they declare that they have no known competing financial interests or personal relationships that could have appeared to influence the work reported in this paper.
